# Overexpression of miR‐516a‐5p Promotes Erosive Oral Lichen Planus: In Vitro Study Based on Bioinformatics Analyses

**DOI:** 10.1002/cre2.70270

**Published:** 2025-12-28

**Authors:** Yan Chen, Chen‑xi Li, Rui Xue, Zhao‐xia Cong, Yi Bian, Yuan Liu

**Affiliations:** ^1^ Division of Pediatric Dentistry and Preventive Dentistry, School/Hospital of Stomatology The First Affiliated Hospital of Xinjiang Medical University Urumqi China; ^2^ Department of Oral and Maxillofacial Oncology & Surgery, School/Hospital of Stomatology, The First Affiliated Hospital of Xinjiang Medical University National Clinical Medical Research Institute Urumqi China; ^3^ Stomatological Research Institute of Xinjiang Uygur Autonomous Region Urumqi China; ^4^ Department of Oral Medicine, School/Hospital of Stomatology The First Affiliated Hospital of Xinjiang Medical University Urumqi China; ^5^ Department of Cariology and Endodontics, School/Hospital of Stomatology, The First Affiliated Hospital of Xinjiang Medical University National Clinical Medical Research Institute Urumqi China; ^6^ Unit of Dermatology and Mucosa and Pathology The First Affiliated Hospital of Xinjiang Medical University Urumqi China

**Keywords:** cytokines, erosive oral lichen planus, human oral mucosal fibroblasts, miR‐516a‐5p, T helper cell

## Abstract

**Objectives:**

This study aimed to investigate the differentially expressed microRNAs in erosive oral lichen planus, followed by analyzing how the overexpression of identified miR‐516a‐5p influences human oral mucosal fibroblasts.

**Material and Methods:**

High‐throughput sequencing using tissues from patients and healthy individuals identified varying microRNA expression profiles in erosive oral lichen planus tissues. Bioinformatics analysis subsequently revealed the enriched pathways and targeted genes involved. In vitro experiments were performed to confirm the validity of bioinformatic findings.

**Results:**

A total of 82 microRNAs were differentially expressed in erosive oral lichen planus tissues. These microRNAs are mainly linked to T helper cell differentiation that enriched in MAPK signaling pathways, as well as targeting mRNAs like MAPK11. Overexpression of miR‐516a‐5p resulted in a statistically significant decrease in MAPK11 mRNA level in human oral mucosal fibroblasts. Overexpression of miR‐516a‐5p increased TNF‐α and IFN‐γ levels, whereas it decreased IL‐4, IL‐6, IL‐10, and IL‐13 levels. Overexpression of miR‐516a‐5p enhanced both the proliferation and migration of human oral mucosal fibroblasts.

**Conclusions:**

The miR‐516a‐5p overexpression in patients with erosive oral lichen planus may contribute to the imbalance of T helper 1/2 cell‐associated inflammatory cytokine expression in human oral mucosal fibroblasts by targeting MAPK11 mRNA, promoting their proliferative and migratory capacities.

## 1 Introduction

Oral lichen planus (OLP), a chronic inflammatory condition affecting 0.1%–4% of the population, predominantly targets the buccal mucosa, tongue, and gingiva in middle‐aged women (Hamour et al. 2020). Erosive OLP (eOLP) is a severe form that leads to epithelial barrier damage, causing intense pain and significant physical and mental distress. Chronic erosive lesions, particularly (eOLP), carry a risk of malignant transformation, leading the World Health Organization to classify it as a precancerous condition (de Lanna et al. 2022). However, the etiology and pathogenesis of eOLP remain unclear, possibly due to immune dysfunction, psychological stress, infections, or local irritants (Li et al. 2023, 2022). At the moment, a definitive curative approach for OLP has not been identified. The current treatment modalities predominantly involve the local or systemic administration of glucocorticoids, immunomodulatory agents, and the supplementation of trace elements (Polizzi et al. 2025). Notably, a recent study revealed that some microRNAs (miRNAs) were correlated with OLP extent and were significant predictors of OLP symptoms, signs, and disease severity scores (Polizzi et al. 2024).

MiRNAs are endogenous non‐coding RNAs approximately 22 nucleotides that regulate target gene expression post‐transcriptionally, influencing processes such as cell development, proliferation, and metabolism (Li, Su, et al. 2024). Their involvement extends to the development of various diseases, including cardiovascular diseases, leukemia, and tumors (Prado et al. 2023; Saenz‐Pipaon and Dichek 2023; Li et al. 2020). As a result, miRNA‐based gene therapy has become a significant focus of global research; studies have indicated that miRNAs may promote the development of OLP through the regulation of epithelial cell apoptosis (Wang et al. 2023; Didona et al. 2022). Research demonstrates that the differentiation of T helper (Th) cells within the lamina propria is a key factor in the development of eOLP (El‐Howati et al. 2023; Li et al. 2023; Wang et al. 2016). Fibroblasts primarily reside within the lamina propria of mucosal tissues, forming the major stromal cell population in the connective tissue matrix. These cells play a crucial role in mediating immune cell recruitment and transendothelial migration into the stromal compartment. Following immune cell infiltration, fibroblasts initiate specific intracellular signaling cascades and ligand‐receptor interactions, which subsequently facilitate immune cell trafficking toward the epithelial border region and contribute to epithelial cell cytotoxicity (Li et al. 2023). Although researchers have investigated miRNA expression in OLP using advanced sequencing techniques, there is still limited research on their functions and mechanisms in eOLP subtype. eOLP is characterized by epithelial barrier disruption, intense pain, and pronounced inflammatory responses, suggesting that its pathogenesis may significantly differ from that of non‐erosive OLP (de Lanna et al. 2022). Therefore, this study aims to identify miRNAs specifically associated with the erosive phenotype, thereby elucidating their unique roles in the development and progression of eOLP.

We accordingly employed high‐throughput sequencing technology to perform bioinformatics analyses based on samples of oral mucosal tissue which were collected from erosive and non‐erosive OLP patients and healthy individuals. We specifically focused on the identified differentially expressed miRNAs contributing to the development and progression of eOLP. In vitro experiments were conducted on the screened miRNAs to validate their potential roles in eOLP, aiming to identify specific biomarkers and therapeutic targets for eOLP.

## 2 Materials and Methods

The study protocol was reviewed and approved by the ethics committee of authors' affiliation (approval no. K202304‐11). Procedures in this research adhered to the standards of the Helsinki Declaration and laboratory regulations in China. All data collected during this study are included in this published article. All participating individuals provided informed consent.

### 2.1 Patient Selection

The study involved patients with eOLP, who were primarily diagnosed on the basis of their medical history, clinical signs and symptoms, and histopathological biopsy reports, according to the World Health Organization's diagnostic criteria (van der Meij and van der Waal 2003).

To be included in this study, patients had to meet the following criteria: (i) The lesions located in the gingiva, tongue, buccal mucosa, hard and soft palate, present as distinct white and gray‐white stripes, along with small papules. (ii) The pathological biopsy manifestations included marked hyperkeratosis of the epithelium, liquefaction of basal cells, and dense infiltration of lymphocytes in intrinsic layer (Zhang et al. 2022). Patients who met any of the following criteria were excluded: (i) Smokers and patients with severe alcohol use disorder. (ii) Patients were diagnosed with other oral mucosal diseases. (iii) The affected regions in patients exhibit dental restorations and biomaterials that correspond anatomically to the lesion locations. (iv) Patients with tumors, severe systemic diseases, and other autoimmune diseases, experienced a profound decline in their quality of life. (v) Patients received organ transplantation, or immunomodulators/preparations within 3 months. (vi) Females were in the period of menstruation, pregnancy, or lactation.

### 2.2 Sample Collection

Oral mucosal tissues were obtained from 10 patients who suffered with OLP, including 5 with erosive and 5 with non‐erosive forms respectively, at the Department of Oral Medicine, School/Hospital of Stomatology, The First Affiliated Hospital of Xinjiang Medical University. The samples from lesion area were sectioned into two parts measuring 3 × 6 mm, respectively. One piece was used for histopathological examination, while the other was preserved at −80°C in an RNase‐free solution (ThermoFisher Scientific). Five cases of healthy individuals seeking removal of impacted teeth in the lower jaw were collected to serve as control subjects in the subsequent experiment. A piece of gingival mucosal tissue approximately 2 × 4 mm in size, covering on the upper part of the blocked teeth, was cut and stored at −80°C for spare. The information of human tissue samples collected in this study is presented in Table 1. A flow diagram describing the subjects' enrollment as well as the working plan is given in Figure S1 in the Supporting Information Appendix.

**Table 1:** Information on human tissue sample collection.

Categories	Sampling sites	Sex	Age
EO #1	Right buccal mucosa	Male	61
EO #2	Right buccal mucosa	Male	52
EO #3	Left ventral tongue mucosa	Female	55
EO #4	Left buccal mucosa	Female	60
EO #5	Left buccal mucosa	Female	64
NEO #1	Right buccal mucosa	Female	57
NEO #2	Left buccal mucosa	Female	62
NEO #3	Left buccal mucosa	Male	54
NEO #4	Right ventral tongue mucosa	Female	55
NEO #5	Left ventral tongue mucosa	Female	59
N #1	The gingival mucosa covering the 38 tooth	Female	50
N #2	The gingival mucosa covering the 38 tooth	Male	45
N #3	The gingival mucosa covering the 48 tooth	Female	47
N #4	The gingival mucosa covering the 48 tooth	Female	55
N #5	The gingival mucosa covering the 48 tooth	Male	49

Abbreviations: EO = erosive oral lichen planus (eOLP), N = normal oral mucosa, NEO = non‐erosive OLP.

### 2.3 High‐Throughput Sequencing Preparation

The tissue samples from 15 specimens were ground using a high‐throughput tissue grinder (Retsch Verder Group) to obtain total RNA extracts. Agarose gel electrophoresis was employed to detect RNA degradation and contamination, and RNA purity was quantified using a Nanodrop microvolume spectrophotometer (ThermoFisher Scientific). The RNA concentration was quantified with a Qubit 4 fluorometer (ThermoFisher Scientific). Additionally, RNA integrity was confirmed using an Agilent 2100 bioanalyzer (Agilent Technologies). After qualifying the samples, the NEBNext Small RNA (NEW ENGLAND Biolabs) was used to construct the library and the Agilent 2100 for assessment. To maintain high quality, reverse transcription‐quantitative polymerase chain reaction (RT‐qPCR) was used to determine the library's effective concentration accurately. Once the library inspection was approved, various libraries were processed on the NovaSeq. 6000 sequencing platform (Illumina Inc.) according to their effective concentration and the required target data volume.

### 2.4 Bioinformatics Analyses of miRNAs

Initially, we de‐junctioned and quality‐filtered the raw sequencing data. We then employed miRDeep2 software package (https://github.com/rajewsky-lab/mirdeep2) for genome comparison to identify known miRNAs and analyzed their secondary structures. This analysis revealed the characteristic hairpin structure of miRNA precursors, which facilitated the prediction of new miRNAs. Finally, we quantified the expression levels of both known and new miRNAs in each sample.

After identifying the differentially expressed miRNAs among the comparisons of each group through DESeq. 2 software (https://www.huber.embl.de/users/anders/DESeq. 2/), with a significance threshold set at *p* < 0.05, we conducted KEGG (Kyoto Encyclopedia of Genes and Genomes) enrichment analysis on the target genes related to these miRNAs in each group based on their known interactions.

### 2.5 Cell Culturing and Immunofluorescence (IF) Evaluation

The human oral mucosal fibroblasts (hOMFs) cell line was provided by Biobank of Oral Medicine and Pathology, Central Scientific and Research Institute of Stomatology, Xinjiang, China (Li, Li, et al. 2024). The cell line was maintained in Dulbecco's Modified Eagle Medium (DMEM, ThermoFisher Scientific) that supplemented with 10% fetal bovine serum (FBS, ThermoFisher Scientific), 1% endothelial growth supplement (Sigma‐Aldrich) and antibiotics—100 µg/mL penicillin and streptomycin (ThermoFisher Scientific) at 37°C with 5% CO_2_. The cells were digested with trypsin‐EDTA (Merck KGaA) and passaged into a T25 culture flask for subsequent experiments. Cell cultures between the third and eighth passages were used in this study (Wang et al. 2020). To be identified, fluorescent staining of hOMFs was performed with anti‐Vimentin antibody (1:200) (Sigma‐Aldrich), and nuclei were marked by 4′,6‐Diamidine‐2′‐phenylindole dihydrochloride (DAPI, Sigma‐Aldrich) (Figure S2 in the Supporting Information Appendix).

### 2.6 Lentiviral Transfection of hOMFs With Overexpressed miR‐516a‐5p

Two or third‐generation hOMFs grown well were divided into three groups: (i) overexpression miR‐516a‐5p lentivirus group (LV‐miR‐516a‐5p), (ii) negative control lentivirus group (miR‐NC), and (iii) blank control group (control). The lentivirus was purchased from GeneChem Biotech Inc. The hOMFs were transfected under optimal conditions with a multiplicity of infection (MOI) of 20, as determined in the pre‐experiment. The cells were incubated with lentivirus for 48 h and screened with 5 μg/mL puromycin (ThermoFisher Scientific). The transfection efficiency and cell growth status were observed using an inverted fluorescence microscope after 72 h of culture.

### 2.7 Flow Cytometry

The hOMFs were seeded in six‐well plates at 1.5 × 10^6^ cells and placed in an incubator until the cells were 90% confluent. Removed the cell culture solution, washed the cells with phosphate buffered saline (PBS) three times, and added the enhanced green fluorescent protein (eGFP. Abcam). The fluorescence intensity was measured by flow cytometer for automatic computer analysis.

### 2.8 qRT‐PCR

Total RNA was extracted from hOMFs in each group utilizing TRIzol reagent (Invitrogen) as directed. Reverse transcribe 1 μg of total RNA into complementary DNA with the KiCqStart One‐Step Probe RT‐qPCR ReadyMix (Sigma‐Aldrich). SYBR Green I method using Premix Ex Taq II (Takara Bio Inc.) was performed by the Applied Biosystems 7500 Real‐Time Quantitative Polymerase Chain Reaction System to measure expression levels. The comparative threshold (*C*_t_) values for the reference and the target were recorded using the results of internal referring standardization (Table S1 in the Supporting Information Appendix). GAPDH was utilized as an endogenous control for data analysis. The raw data were calculated according to the Livak formula (comparative threshold cycle value [2^−ΔΔCt^]). The sequence of the primers used in this study is listed in Table 2.

**Table 2:** PCR primer sequence in this study.

Primer name	Sequence (5" to 3")	Fragment (bp)
miR‐516a‐5p	F: GAGGAGGAGGAAGAGGAAGACAGAG	24
	R: GACGGTCATAGCCCAATCCCTTTG	
MAPK11	F: GGAGCAGCAAGAGCAAGGAGAAG	23
	R: TCAGTGGACAGTAGAACGGAGGAAAG	

Abbreviation: bp = base pair.

### 2.9 Enzyme‐Linked Immunosorbent Assay (ELISA)

Once the cell cultures reached approximately 90% confluence, the medium was replaced, and the cells were cultured for another 3 days. The supernatant from each group was collected and centrifuged at 4000 rpm for 20 min. Subsequently, the concentrations of TNF‐α, IFN‐γ, IL‐6, and IL‐10 were detected following the instructions provided in the ELISA kits. Each sample was run in triplicate.

### 2.10 Cell Counting Kit‑8 (CCK‑8) Assay

The CCK‐8 (Abcam) assay was used to assess cell proliferation in hOMFs. The cells were plated into 96‐well plates at a density of 5 × 10^3^ cells per well. Each group of cells was first digested and centrifuged for counting; then, 1000 cells were seeded into 96‐well plates per well, with six replicates for every group. Subsequently, 10 µL of CCK‐8 solution was added to each well, and the cells were incubated for 2 h at 37°C. The absorbance was detected at a wavelength of 450 nm by a microplate reader (Tecansunrise), and the difference of cell viability between groups was analyzed at 1.5 h.

### 2.11 Scratch Wound Healing Assay

The migration ability of hOMFs from each group was assessed in wound healing scratch assay. The cells were seeded (5 × 10^5^) in 6‐well plates and scratched by a sterile 200 μL pipette tip when the cells reached 90% confluency. The width of the wound was photographed and measured at 0 h, 24 h, and 48 h, respectively. Cell migration was analyzed using ImageJ version 1.8.0 software (National Institutes of Health).

### 2.12 Statistical Analyses

Experimental data were analyzed using Statistical Package for Natural Science version 26.0 (IBM SPSS). The Shapiro–Wilk test was used to verify the normality of all data. Normally distributed data are expressed as mean ± standard deviation (SD) ($\bar{x}\pm s$); and one‐way analysis of variance was performed to compare groups. The Kruskal–Wallis H test was employed for the non‐normal data. A *p* value less than 0.05 was considered statistically significant. GraphPad Prism software version 9.5 (Graph Pad Software Inc.) was used to plot values.

## 3 Results

### 3.1 Identification of Differentially Expressed miRNA in eOLP Tissue

**Figure 1:**
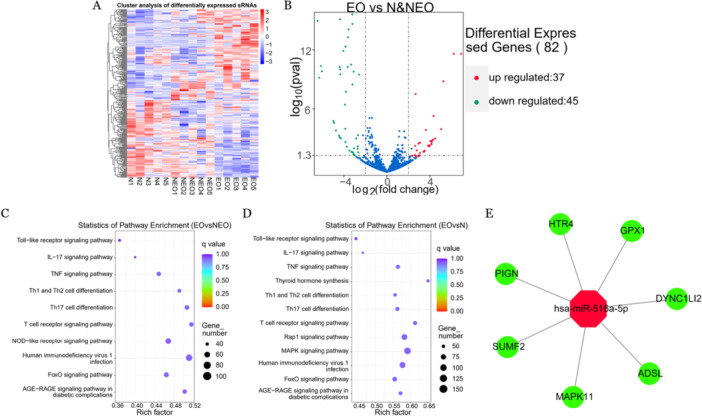
Bioinformatics analyses of high‐throughput sequencing data. (A) Heatmap of the total differentially expressed miRNAs based on the value of |log_2_ FC| > 2, adj. *p* < 0.05. (B) The red points in the volcano plot represent upregulation and the green plots represent downregulation. KEGG enrichment was shown using a bubble diagram. (C) EO group versus NEO group (D) EO group versus N group. Colors represent the *p* values and sizes of the bubbles represent the counts of genes. (E) Target gene prediction of miR‐516a‐5p computed by MCC algorithm. adj. = adjusted, EO = erosive oral lichen planus (eOLP), FC = fold change, KEGG = Kyoto Encyclopedia of Genes and Genomes, MCC = maximum correntropy criterion, N = normal oral mucosa, NEO = non‐erosive OLP.

After denoising and standardizing the expression profile of sequencing datasets, the distribution of differential miRNA expression between samples from three groups (eOLP, non‐erosive OLP, and normal oral mucosa) was visualized by heatmap and volcano plot (Figure 1A,B). A total of 82 differentially‐expressed miRNAs were identified, including 37 upregulated miRNAs and 45 downregulated miRNAs.

Bayesian cluster analysis revealed significantly upregulated and downregulated miRNAs in eOLP tissue, indicating miR‐516a‐5p showing the most notable upregulation with |log_2_(Fold Change)| = 6.48 and *p* < 0.001 (Table 3). The top 10 significantly differentially‐expressed miRNAs are listed in Table 3.

**Table 3:** Significantly up‐ and down‐regulated miRNAs in erosive oral lichen planus.

miRNA name	log_2_FC value	*p* value	Expression
miR‐516a‐5p	6.48	< 0.001	Up‐regulated
miR‐526b‐5p	5.87	< 0.001	Up‐regulated
miR‐517a‐3p	4.50	< 0.001	Up‐regulated
miR‐517b‐3p	4.50	< 0.001	Up‐regulated
miR‐517c‐3p	4.32	< 0.001	Up‐regulated
miR‐519d‐3p	3.84	< 0.001	Up‐regulated
miR‐516b‐5p	3.71	< 0.001	Up‐regulated
miR‐518a‐3p	3.67	< 0.001	Up‐regulated
miR‐518e‐3p	3.59	< 0.001	Up‐regulated
miR‐548c‐5p	3.58	< 0.001	Up‐regulated
miR‐211‐5p	−6.13	< 0.001	Down‐regulated
miR‐506‐3p	−5.82	< 0.001	Down‐regulated
miR‐375‐3p	−5.31	< 0.001	Down‐regulated
miR‐509‐5p	−5.23	< 0.001	Down‐regulated
miR‐6510‐3p	−4.29	< 0.001	Down‐regulated
miR‐23a‐5p	−4.23	< 0.001	Down‐regulated
miR‐6512‐5p	−4.09	< 0.001	Down‐regulated
miR‐200c‐5p	−3.84	< 0.001	Down‐regulated
miR‐200a‐3p	−3.79	< 0.001	Down‐regulated
miR‐1468‐5p	−3.79	< 0.001	Down‐regulated

Abbreviation: FC = fold change.

**Figure 2:**
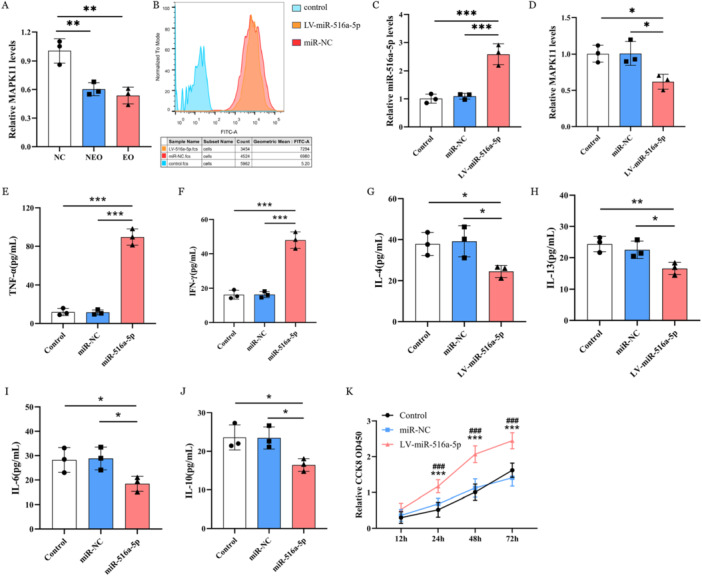
(A) Through RT‐qPCR analysis, we quantitatively assessed MAPK11 mRNA expression profiles in OLP tissue specimens. (B) Flow cytometry fluorescence detection of transfection efficiency in hOMFs using EGFP. (C,D) RT‐PCR method performed to detect the expression levels of miR‐516a‐5p (C) and its target gene MAPK11 (D) in hOMFs. (E–J) ELISA approach used to measure the concentration of (E) TNF‐α, (F) IFN‐γ, (G) IL‐4, (H) IL‐13, (I) IL‐6, and (J) IL‐10, respectively. (K) CCK‐8 assay showed the effect of overexpression of miR‐516a‐5p on proliferation of hOMFs. Differences were considered significant if *p < 0.05, **p < 0.01, ***p < 0.001. ns, *p* > 0.05. CCK‑8 = cell counting kit‑8, EGFP = enhanced green fluorescent protein, ELISA = enzyme‐linked immunosorbent assay, EO = erosive oral lichen planus (eOLP), hOMFs = human oral mucosal fibroblasts, LV = lentivirus vector, NC = negative control, NEO = non‐erosive OLP, OD = optical density, RT‐qPCR = reverse transcription‐quantitative polymerase chain reaction.

Functional enrichment of these differentially‐expressed miRNAs and prediction of their associated target genes were carried out through miRanda online tool (https://www.microrna.org/) and TargetScan (https://www.targetscan.org/) database, followed by KEGG analysis (Figure 1C,D). Seven hub genes that miR‐516a‐5p targeted were obtained by the maximum correntropy criterion (MCC) algorithm (Figure 1E). Possible targets of miR‐516a‐5p action included the mRNA of mitogen‐activated protein kinase 11 (MAPK11) of the MAPK signaling pathway. The results of RT‐qPCR analysis indicated that the expression level of MAPK11 mRNA was significantly downregulated in the NEO and EO tissue samples (*p* < 0.01) (Figure 2A).

### 3.2 Transfection of Lentiviral Vectors for Stable hOMFs Overexpressing miR‐516a‐5p

**Figure 3:**
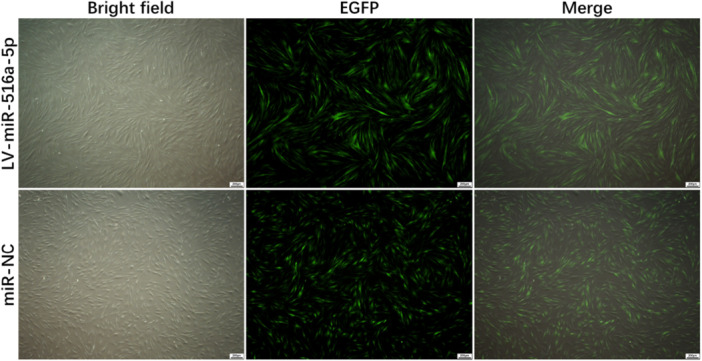
The expression of EGFP in the cells of the LV‐miR‐516a‐5p group and miR‐NC group was observed under an inverted fluorescence microscope after transfection of lentiviral vectors into hOMFs for 72 h (original magnification × 100). EGFP = enhanced green fluorescent protein, hOMFs = human oral mucosal fibroblasts, LV = lentivirus, NC = negative control.

The miR‐NC and miR‐516a‐5p lentiviruses were transfected into a hOMFs cell model. After a 72‐h transfection of lentiviral vectors into hOMFs, we used an inverted fluorescence microscope to observe eGFP expression in the cells of the LV‐miR‐516a‐5p and miR‐NC groups (Figure 3). The successful transfection of miR‐516a‐5p and miR‐NC was confirmed, and its transfection efficiency was visualized via flow cytometry (Figure 2B). The eGFP labeling rates for both the LV‐miR‐516a‐5p group and the miR‐NC group exceeded 95%, and the difference in labeling rates between these two groups was not statistically significant (*p* > 0.05). As a result, hOMFs that overexpress miR‐516a‐5p are suitable for further experimental studies.

### 3.3 Effect of miR‐516a‐5p Overexpression Targeted MAPK11

The results of RT‐qPCR, detected the expression levels of miR‐516a‐5p along with its target gene MAPK11, indicated that the expression level of miR‐516a‐5p was significantly higher in the LV‐miR‐516a‐5p group than that in miR‐NC and control groups (*p* < 0.001) (Figure 2C); whereas the MAPK11 mRNA expression was significantly downregulated in the LV‐miR‐516a‐5p group compared with the miR‐NC and control groups (*p* < 0.01) (Figure 2D). The difference of miR‐516a‐5p and MAPK11 mRNA expression levels lies between the miR‐NC group and the blank control group was not statistically significant (all *p* > 0.05) (Figure 2C,D). The findings suggest that miR‐516a‐5p definitively targets MAPK11 mRNA and inhibits its expression level.

### 3.4 Impact of miR‐516a‐5p Overexpression on Th1/Th2 and Th17/Treg Cell‐Related Inflammatory Cytokines Secretion

Given Th1 and Th2 cell differentiation was annotated as predominantly enriched biological processes targeted by miR‐516a‐5p in eOLP tissue, we determined the secretion of TNF‐α, IFN‐γ, IL‐4, and IL‐13 which associated with Th1/2 cell differentiation. The expression levels of Th1‐related inflammatory cytokines TNF‐α and IFN‐γ were significantly higher in the LV‐miR‐516a‐5p group than in both the miR‐NC and blank control groups (all *p* < 0.001) (Figure 2E,F). However, there was no statistically significant difference between the miR‐NC group and the blank control group (*p* > 0.05) (Figure 2E,F). In contrast, in the LV‐miR‐516a‐5p group, Th2‐related cytokines IL‐4 and IL‐13 were significantly reduced (all *p* < 0.05), while no significant differences were found between the miR‐NC and control groups (*p* > 0.05) (Figure 2G,H). These results indicate that overexpressed miR‐516a‐5p contributed to an imbalance of Th1/Th2 inflammatory cytokine expression in hOMFs. Additionally, we found that the expression levels of IL‐6 which is associated with Th17 cell differentiation, and IL‐10 which is related to Treg cell differentiation, were significantly decreased in the LV‐miR‐516a‐5p group (all *p* < 0.05). However, there were no significant differences between the miR‐NC group and the control group (*p* > 0.05) (Figure 2I,J). Therefore, the overexpressed miR‐516a‐5p in eOLP tissues may also, to some extent, affect the differentiation of Th17 and Treg cells.

### 3.5 Impact of miR‐516a‐5p Overexpression on hOMF Proliferation

Growth curves illustrated that the proliferative capability of hOMFs increased significantly across all groups, with the LV‐miR‐516a‐5p group demonstrating superior activity compared to both the miR‐NC and blank control groups (all *p* < 0.001) (Figure 2K). Conversely, the difference in proliferative activity between the hOMFs in the miR‐NC group and the blank control group was not statistically significant (all *p* > 0.05) (Figure 2K). The results demonstrated that the overexpression of miR‐516a‐5p effectively promotes the proliferative capacity of hOMFs.

### 3.6 Impact of miR‐516a‐5p Overexpression on hOMF Migration

**Figure 4:**
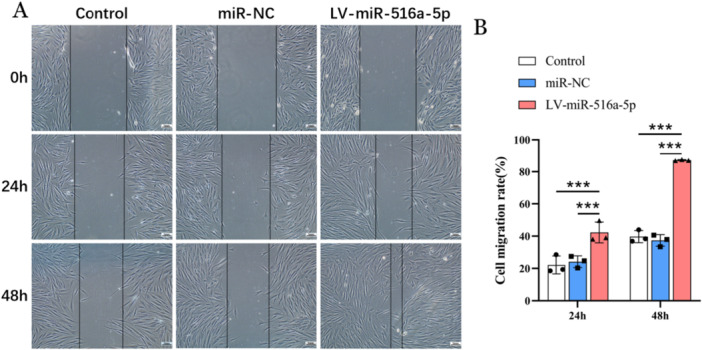
Effect of overexpression of miR‐516a‐5p on migration of hOMFs. (A) Scratch wound healing assay at 0 h, 24 h, and 48 h, respectively. (B) Quantitative histogram of Figure 4A. Differences were considered significant if ***p < 0.001. ns, *p* > 0.05. hOMFs = human oral mucosal fibroblasts, LV = lentivirus vector, NC = negative control.

Significantly increased activation of migration was observed in hOMF cells of LV‐miR‐516a‐5p group at 24 h and 48 h, respectively (all *p* < 0.001) (Figure 4A,B). At both 24 and 48 h, there was no statistically significant difference in the wound healing rate between the LV‐miR‐NC group and the blank control group (all *p* > 0.05) (Figure 4A,B). The results demonstrated that the miR‐516a‐5p overexpression enhances the migratory capacity of hOMFs.

## 4 Discussion

Our preliminary investigations suggest that the overexpression of miR‐516a‐5p affects the progression of erosive lesions in OLP. However, the causes and mechanisms for OLP progression are still not fully understood, and the disease has no definitive treatment either. Treatment mainly includes topical or systemic corticosteroids, immunomodulatory drugs, and micronutrient supplements (Louisy et al. 2024; Lodi et al. 2020). Critical therapeutic goals are to reduce the psychological burden on patients, improve their quality of life, decrease cancer risk, and promote the healing of eOLP lesions to prevent recurrence. The histopathological features of OLP consist of hyperkeratosis of the epithelium, changes in the spinous layer (either hyperplasia or atrophy), basal cell liquefaction, and lymphocytic infiltration, which can be band‐like or focal in the superficial lamina propria (Deng et al. 2023). Numerous studies have shown that T lymphocytes significantly mediate local immune responses, which are crucial to the pathogenesis of OLP (Deng et al. 2023; Qing et al. 2023; Wang et al. 2016). Wang et al. indicated that CD4^+^ T cells are the primary type of lymphocytes found in the mucosal epithelium and lamina propria of OLP lesions (Wang et al. 2016). These cells can differentiate into various Th subsets, including Th1 and Th2, when stimulated by specific inflammatory cytokines (Shang et al. 2024). It is therefore essential to investigate the role of T lymphocytes in eOLP lesions to better explore risk factors and give proper interventions that would greatly benefit individuals suffering from this chronic condition.

In recent years, as next‐generation sequencing technique has rapidly developed, targeted re‐sequencing in particular gene panel sequencing is becoming more and more popular in medical genetics, both for research projects and in diagnostic settings (Altmüller et al. 2014). Besides, the regulatory roles of various non‐coding RNAs, including miRNAs, in the development of cardiovascular diseases, tumors, and other conditions have attracted widespread attention (Qiu et al. 2021). For instance, many studies have demonstrated that miR‐516a‐5p can be involved in the progression of hepatocellular carcinoma (Liu et al. 2022; Chen et al. 2021). Especially, Yao et al. (2019) discovered that MAPK11 is related to the development of liver cancer and concurrently confirmed that MAPK11 is a target gene of miR‐516a‐5p. However, the function of miRNAs in eOLP occurrence and progression still needs further investigation. A latest systematic review and meta‐analysis discovered that miR‐27a/b, miR‐142, miR‐137, miR‐1246, miR‐1290, and miR‐4484 had the highest diagnostic odds ratio for OLP, based on miRNAs expression profiling (Koopaie et al. 2024). According to Ge et al. (2020) miRNA‐27b regulates the abnormal proliferation and apoptosis of oral mucosal epithelial cells, which contributes to OLP development. Another study uncovered that miRNA‐122 is elevated in the epithelial layer of OLP lesions, leading to keratinocyte apoptosis and a disrupted epithelial barrier by interfering with mRNA expression targeting vitamin D receptor (Ge et al. 2021). Therefore, detailed investigation of miRNA expression patterns and their biological roles in eOLP lesional tissues provides crucial insights for understanding disease mechanisms and identifying potential therapeutic targets.

In this study, we investigated the potential role of miRNAs in the development of eOLP through bioinformatics analysis of eOLP tissues applying high‐throughput sequencing technology for the first time. We screened and identified 37 miRNAs that were significantly upregulated and 45 that were downregulated totally. Among these, miR‐516a‐5p was identified as one of the most significantly upregulated miRNAs. In addition, clustering enrichment analysis showed that these miRNAs mainly contribute to Th1 and Th2 cell differentiation, with their target genes linked to several signaling pathways, such as MAPK, IL‐17, and Toll‐like receptors. Notably, miR‐516a‐5p targets the mRNA of MAPK11, a key component of the MAPK signaling pathway. A few studies have previously reported that MAPK11 functions as an important point of integration in signaling transduction pathways and controlling endocellular processes, including viability of cells, differentiation, proliferation and apoptosis (Song et al. 2023; Liu et al. 2020; Li et al. 2019). Our in vitro cell experiments confirmed that increasing miR‐516a‐5p levels in hOMFs diminishes MAPK11 mRNA expression. This reduction disrupts the balance of Th1 and Th2 inflammatory cytokines and encourages the proliferation and migration of hOMFs. Interestingly, our study revealed that increased expression levels of miR‐516a‐5p promoted the proliferation and migration of hOMFs, suggesting that miR‐516a‐5p may be involved in tissue remodeling and fibrosis, which are common features of eOLP. Since fibroblast migration is a critical step in wound healing and tissue repair, excessive migration may lead to pathological changes such as fibrosis and chronic inflammation (Li et al. 2019).

However, this study presents several methodological constraints, particularly the absence of single‐cell resolution in genomic sequencing analyses. The lack of single‐cell omics data may limit the detection of cellular heterogeneity and rare cell populations within the studied samples. Future studies incorporating single‐cell RNA sequencing or single‐nucleus approaches would provide more comprehensive insights into cell‐type‐specific molecular signatures. Such technical advancements could significantly enhance the resolution and biological relevance of the findings. Our research was solely focused on the functional investigation of miR‐516a‐5p in hOMFs. In fact, it is of great significance to study the expression of miR‐516a‐5p in other cell types such as macrophages and epithelial cells. Different cell types play distinct roles in the pathogenesis of OLP. miR‐516a‐5p may exert its functions in multiple cell types, influencing cell‐cell interactions and the disease progression. Exploring the expression of miR‐516a‐5p in other cell types and further clarifying its comprehensive role in the pathogenesis of OLP will contribute to a deeper understanding of the disease development process and provide a more comprehensive theoretical basis for identifying more effective therapeutic targets.

## 5 Conclusion

In summary, this study used high‐throughput sequencing and bioinformatics to analyze oral mucosal tissues from patients with eOLP, identifying differentially expressed miRNAs, especially miR‐516a‐5p. Our experimental results demonstrated that miR‐516a‐5p plays a crucial role in disrupting the equilibrium between Th1 and Th2 cytokine profiles, while simultaneously stimulating the proliferative and migratory capacities of hOMFs. These findings suggest its potential involvement in the disease progression of eOLP. This research lays the groundwork for further investigation into the regulatory roles of miRNAs in OLP development and may help identify potential therapeutic targets for this condition. The findings of this study suggest that subsequent investigations should incorporate comprehensive multi‐omics approaches using clinical samples, coupled with the development of appropriate animal models, to thoroughly characterize the molecular pathways influenced by miR‐516a‐5p and confirm its biological significance under physiological conditions.

## Author Contributions

Conceptualization: Chen‐xi Li and Yan Chen. Writing – original draft: Yan Chen and Chen‐xi Li. Writing – revision, review and editing: Chen‐xi Li. Data curation: Yan Chen and Rui Xue. Formal analysis: Chen‐xi Li, Yan Chen, and Zhao‐xia Cong. Funding acquisition: Chen‐xi Li and Yuan Liu. Investigation: Yan Chen, Yuan Liu, Rui Xue, Zhao‐xia Cong, and Yi Bian. Methodology: Chen‐xi Li, Yuan Liu, Rui Xue, and Yi Bian. Supervision: Chen‐xi Li. Validation: Yan Chen, Rui Xue, Zhao‐xia Cong, and Yi Bian. Project administration: Chen‐xi Li and Yuan Liu. All authors read and approved the final manuscript. All authors contributed to the article and approved the submitted version.

## Funding

All phases of this study were supported by the Scientific Research Innovation Project—Hubei Province Key Laboratory of Oral and Maxillofacial Development and Regeneration (grant number: 2022kqhm008); Natural Science Foundation of Xinjiang Uygur Autonomous Region (grant number: 2025D01C175 and 2022D01C253).

## Ethics Statement

The present study was approved by the Ethics Committee of the First Affiliated Hospital of Xinjiang Medical University (approval no. K202304‐11). Procedures operated in this research were completed in keeping with the standards set out in the Announcement of Helsinki and laboratory guidelines of research in China.

## Consent

Written informed consent to participate in this study was provided by the participants or legal guardian/next of kin.

## Conflicts of Interest

The authors declare no conflicts of interest.

## Supporting information


**Supporting Figure 1:** Study flowchart.

**Supporting Figure 2:** The identification of human oral mucosal fibroblasts (hOMFs) by immunofluorescent staining (original magnification × 100). Green (Alexa Fluor 488) indicates cytoplasm. Blue (DAPI) indicates nucleus.

**Supporting Table 1:** Raw data of real‐time quantitative polymerase chain reaction (qRT‐PCR).

## Data Availability

The data that support the findings of this study are available within the manuscript and in the supporting material of this article.
