# Supplementary figures and images for: Overexpression of miR‐516a‐5p Promotes Erosive Oral Lichen Planus: In Vitro Study Based on Bioinformatics Analyses
Source: Clin Exp Dent Res. 2025 Dec 28;11(6):e70270. doi: 10.1002/cre2.70270 (PMC12745659; doi:10.1002/cre2.70270)

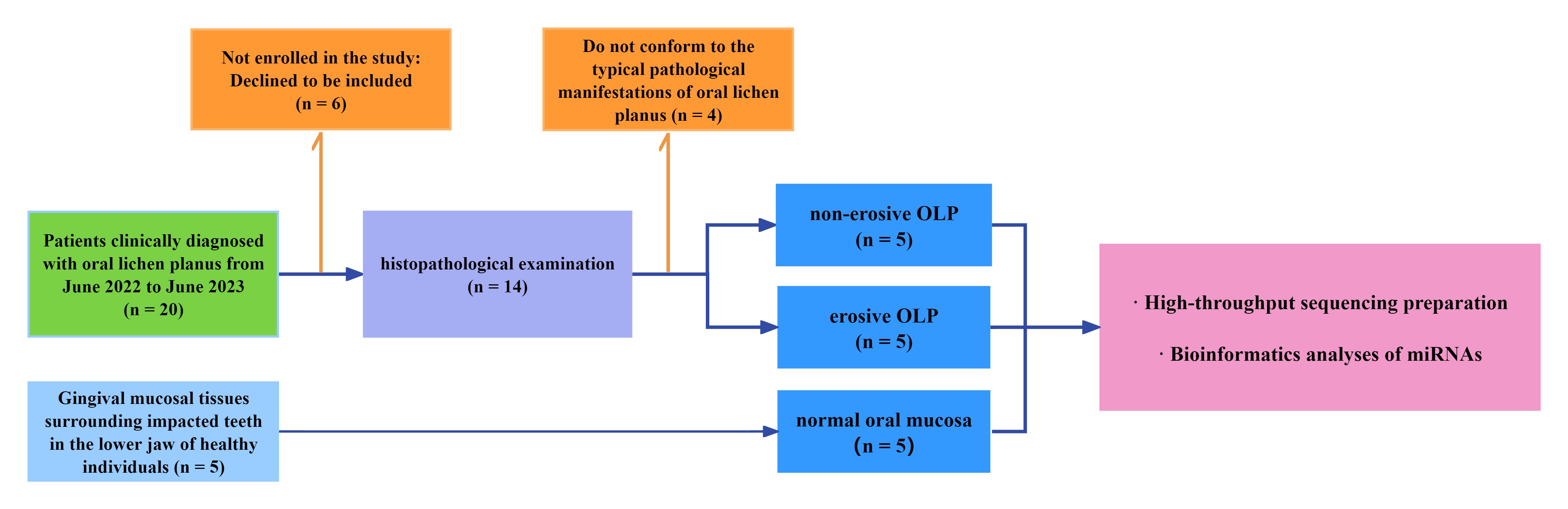

Supplement: Supplementary file 1 — Supporting Figure 1: Study flowchart. [file CRE2-11-e70270-s001.jpg]

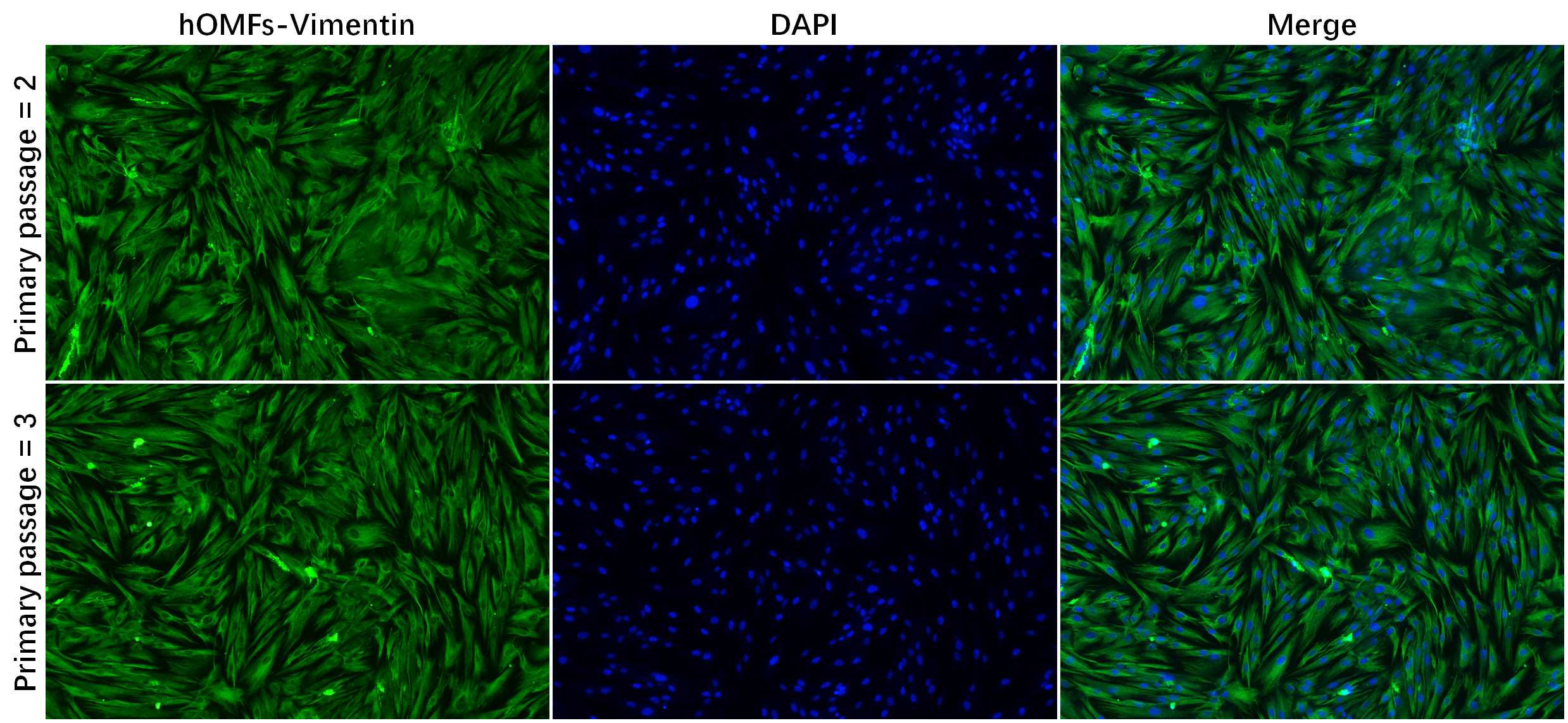

Supplement: Supplementary file 2 — Supporting Figure 2: The identification of human oral mucosal fibroblasts (hOMFs) by immunofluorescent staining (original magnification × 100). Green (Alexa Fluor 488) indicates cytoplasm. Blue (DAPI) indicates nucleus. [file CRE2-11-e70270-s003.tif]
