# Supplementary material for: Overexpression of miR‐516a‐5p Promotes Erosive Oral Lichen Planus: In Vitro Study Based on Bioinformatics Analyses
Source: Clin Exp Dent Res. 2025 Dec 28;11(6):e70270. doi: 10.1002/cre2.70270 (PMC12745659; doi:10.1002/cre2.70270)
Supplement: Supplementary file 3 — Supporting Table 1: Raw data of real‐time quantitative polymerase chain reaction (qRT‐PCR). [file CRE2-11-e70270-s002.docx]

Supplementary Table 1. Raw data of real-time quantitative polymerase chain reaction (qRT-PCR).

|  | Ct | | |  | △ct | |  | Average | |  | △△ct | |  | 2^-△△ct | |
| --- | --- | --- | --- | --- | --- | --- | --- | --- | --- | --- | --- | --- | --- | --- | --- |
| no. | GAPDH | miR-516a-5p | MAPKII |  | miR-516a-5p | MAPKII |  | miR-516a-5p | MAPKII |  | miR-516a-5p | MAPKII |  | miR-516a-5p | MAPKII |
| control | 18.62 | 20.11 | 20.75 |  | 1.49 | 2.13 |  | 1.55 | 2.12 |  | -0.24 | 0.17 |  | 1.18 | 0.89 |
| control | 18.58 | 20.16 | 20.75 |  | 1.58 | 2.17 |  |  |  |  |  |  |  |  |  |
| control | 18.63 | 20.20 | 20.68 |  | 1.57 | 2.05 |  |  |  |  |  |  |  |  |  |
| control | 20.35 | 22.40 | 22.54 |  | 2.05 | 2.19 |  | 2.01 | 1.79 |  | 0.22 | -0.16 |  | 0.86 | 1.12 |
| control | 20.44 | 22.72 | 22.52 |  | 2.28 | 2.08 |  |  |  |  |  |  |  |  |  |
| control | 20.48 | 22.17 | 21.57 |  | 1.69 | 1.09 |  |  |  |  |  |  |  |  |  |
| control | 19.21 | 21.47 | 21.34 |  | 2.26 | 2.13 |  | 1.80 | 1.93 |  | 0.01 | -0.01 |  | 0.99 | 1.01 |
| control | 19.50 | 21.25 | 21.38 |  | 1.75 | 1.88 |  |  |  |  |  |  |  |  |  |
| control | 19.63 | 21.01 | 21.41 |  | 1.38 | 1.78 |  |  |  |  |  |  |  |  |  |
| miR-NC | 19.93 | 21.36 | 22.01 |  | 1.43 | 2.08 |  | 1.58 | 2.10 |  | -0.20 | 0.15 |  | 1.15 | 0.90 |
| miR-NC | 19.87 | 21.49 | 21.98 |  | 1.62 | 2.11 |  |  |  |  |  |  |  |  |  |
| miR-NC | 19.98 | 21.67 | 22.08 |  | 1.69 | 2.10 |  |  |  |  |  |  |  |  |  |
| miR-NC | 22.20 | 22.26 | 23.75 |  | 0.06 | 1.55 |  | 1.57 | 1.69 |  | -0.21 | -0.26 |  | 1.16 | 1.20 |
| miR-NC | 21.93 | 24.31 | 23.65 |  | 2.38 | 1.72 |  |  |  |  |  |  |  |  |  |
| miR-NC | 21.97 | 24.24 | 23.76 |  | 2.27 | 1.79 |  |  |  |  |  |  |  |  |  |
| miR-NC | 19.34 | 21.21 | 21.09 |  | 1.87 | 1.75 |  | 1.82 | 2.05 |  | 0.03 | 0.11 |  | 0.98 | 0.93 |
| miR-NC | 19.12 | 20.91 | 21.22 |  | 1.79 | 2.10 |  |  |  |  |  |  |  |  |  |
| miR-NC | 19.27 | 21.06 | 21.57 |  | 1.79 | 2.30 |  |  |  |  |  |  |  |  |  |
| LV-miR-516a-5p | 21.34 | 21.55 | 24.22 |  | 0.21 | 2.88 |  | 0.24 | 2.93 |  | -1.54 | 0.99 |  | 2.91 | 0.50 |
| LV-miR-516a-5p | 21.38 | 21.69 | 24.34 |  | 0.31 | 2.96 |  |  |  |  |  |  |  |  |  |
| LV-miR-516a-5p | 21.29 | 21.49 | 24.25 |  | 0.20 | 2.96 |  |  |  |  |  |  |  |  |  |
| LV-miR-516a-5p | 20.77 | 21.59 | 23.67 |  | 0.82 | 2.90 |  | 0.65 | 2.56 |  | -1.13 | 0.62 |  | 2.19 | 0.65 |
| LV-miR-516a-5p | 20.64 | 21.56 | 23.23 |  | 0.92 | 2.59 |  |  |  |  |  |  |  |  |  |
| LV-miR-516a-5p | 20.89 | 21.10 | 23.09 |  | 0.21 | 2.20 |  |  |  |  |  |  |  |  |  |
| LV-miR-516a-5p | 20.14 | 20.70 | 22.60 |  | 0.56 | 2.46 |  | 0.37 | 2.45 |  | -1.41 | 0.51 |  | 2.66 | 0.70 |
| LV-miR-516a-5p | 20.28 | 20.67 | 22.78 |  | 0.39 | 2.50 |  |  |  |  |  |  |  |  |  |
| LV-miR-516a-5p | 20.35 | 20.51 | 22.74 |  | 0.16 | 2.39 |  |  |  |  |  |  |  |  |  |
